# Fibroblast Growth Factor Receptor 4 Polymorphism Is Associated with Liver Cirrhosis in Hepatocarcinoma

**DOI:** 10.1371/journal.pone.0122961

**Published:** 2015-04-10

**Authors:** Ming-Jen Sheu, Ming-Ju Hsieh, Whei-Ling Chiang, Shun-Fa Yang, Hsiang-Lin Lee, Liang-Ming Lee, Chao-Bin Yeh

**Affiliations:** 1 Department of Gastroenterology and Hepatology, Chi Mei Medical Center, Tainan, Taiwan; 2 Cancer Research Center, Changhua Christian Hospital, Changhua, Taiwan; 3 School of Optometry, Chung Shan Medical University, Taichung, Taiwan; 4 Institute of Medicine, Chung Shan Medical University, Taichung, Taiwan; 5 School of Medical Laboratory and Biotechnology, Chung Shan Medical University, Taichung, Taiwan; 6 Department of Medical Research, Chung Shan Medical University Hospital, Taichung, Taiwan; 7 Deptartment of Surgery, Chung Shan Medical University Hospital, Taichung, Taiwan; 8 Department of Urology, Wan Fang Hospital, Taipei Medical University, Taipei, Taiwan; 9 Department of Emergency Medicine, School of Medicine, Chung Shan Medical University, Taichung, Taiwan; 10 Department of Emergency Medicine, Chung Shan Medical University Hospital, Taichung, Taiwan; Taipei Medical University, TAIWAN

## Abstract

**Background:**

Fibroblast growth factor receptor 4 (FGFR4) polymorphisms are positively correlated with tumor progression in numerous malignant tumors. However, the association between FGFR4 genetic variants and the risk of hepatocellular carcinoma (HCC) has not yet been determined. In this study, we investigated the potential associations of FGFR4 single nucleotide polymorphisms (SNPs) with HCC susceptibility and its clinicopathological characteristics.

**Methodology/Principal Findings:**

Four SNPs in FGFR4 (rs1966265, rs351855, rs2011077, and rs7708357) were analyzed among 884 participants, including 595 controls and 289 patients with HCC. The samples were further analyzed to clarify the associations between these gene polymorphisms and the risk of HCC, and the impact of these SNPs on the susceptibility and clinicopathological characteristics of HCC. After adjusting for other covariants, HCC patients who carrying at least one A genotype (GA and AA) at rs351855 were observed to have a higher risk of liver cirrhosis compared with those carrying the wild-type genotype (GG) (OR: 2.113, 95% CI: 1.188–3.831). Moreover, the patients with at least one A genotype were particularly showed a high level of alpha-fetoprotein (AFP).

**Conclusions:**

Our findings suggest that genetic polymorphism in FGFR4 rs351855 may be associated with the risk of HCC coupled with liver cirrhosis and may markedly increase the AFP level in Taiwanese patients with HCC. In addition, this is the first study that evaluated the risk factors associated with FGFR4 polymorphism variants in Taiwanese patients with HCC.

## Introduction

Hepatocellular carcinoma (HCC) is one of the malignant neoplasms common for causing cancer-related deaths worldwide, particularly in Asian countries. More than 80% of HCC cases occur in developing countries, particularly Far East and Southeast Asia, with Taiwan having the third highest incidence rate in the world. HCC develops in complex and multistep manners. Multiple risk factors including chronic hepatitis B virus (HBV) or hepatitis C virus (HCV) infection, cirrhosis, carcinogen exposure, excessive alcohol consumption, and a variety of single nucleotide polymorphisms (SNPs) contribute to hepatocarcinogenesis [1–6]. Surgery is a critical treatment modality in the early stage of HCC. Despite advances in therapy, HCC is still characterized as recurrent and usually results in metastasis, which produces poor patient prognoses. Because only 10%-20% of HCCs can be removed completely by surgery, early diagnosis and prevention can increase the survival rate if the underlying molecular mechanism is identified.

Fibroblast growth factor (FGF) was found in pituitary extracts by Armelin in 1973, followed by cow-brain extracts by Gospodarowicz et al. and tested in a bioassay that caused fibroblasts to proliferate. FGFs are multifunctional proteins with a wide variety of effects-they are most commonly mitogens but they have regulatory, morphological, and endocrinal effects with members involved in angiogenesis, wound healing, and embryonic development. In humans, 22 members of the FGF family have been identified, all of which are structurally related signaling molecules. The human fibroblast growth factor receptor (FGFR) family, a subfamily of the receptor tyrosine kinases, comprises 4 family members: FGFR1, FGFR2, FGFR3, and FGFR4 that consist of 3 extracellular immunoglobulin-type domains (D1-D3), a single-span transmembrane domain, and an intracellular split tyrosine kinase domain [7–10]. Enhanced FGFR expression is commonly observed in various types of human malignancies such as breast, ovarian, bladder, pulmonary, and gastric cancers, and rhabdomyosarcoma [9]. Moreover, FGFR4 is an attractive target for HCC progression because the hepatocyte is the only human cell type where FGFR4 is in the predominant isoform of the FGFRs [11–13]. In addition, the liver was reported to have the highest transcript expression of FGFR4 [14, 15]. Therefore, we hypothesized that FGFR4 contributes to the regulation of HCC development and prognosis.

It has been reported that, for FGFR4, the G/A polymorphisms in rs1966265 (Ile10Val), rs2011077, and rs7708357 and the C/T polymorphisms in rs351855 (Gly388 to Arg388) may affect protein expression [16–18]. Furthermore, these polymorphisms have a pathophysiological impact on tumor development in various cancers [19, 20]. In this study, the relationships between these 4 SNPs of FGFR4 and the risk of HCC were investigated, and the impact of these SNPs on the susceptibility and clinicopathological characteristics of HCC were evaluated.

## Materials and Methods

### Patients and specimens

In this study, we recruited 289 patients with HCC at the Chung Shan Medical University Hospital, Taiwan. A diagnosis of HCC was made according to the criteria specified in the national guidelines for HCC detection. For the control group, we randomly chose 595 non-cancer individuals (487 males and 108 females with a mean age of 51.00 ± 14.95 years) who visited those same hospitals that had neither self-reported history of cancer of any sites. Moreover, information regarding tobacco use (smoker vs. nonsmoker) and alcohol consumption (current heavy drinker, defined by CDC criteria as a person consuming an average of more than 2 drinks per day, vs. not current heavy drinker) were obtained using questionnaires. Patients with HCC were clinically staged at the time of diagnosis according to the tumor, node, and metastasis (TNM) staging system of the American Joint Committee on Cancer (2002)[21]. Liver cirrhosis was diagnosed by using liver biopsy, abdominal sonography, or biochemical evidence of parenchymal damage in the liver with endoscopic esophageal or gastric varices. The patients’ clinicopathological characteristics included clinical staging, tumor size, lymph node metastasis, distant metastasis, presence of the HBV surface antigen (HBsAg), reactivity with antibody against HCV (anti-HCV), liver cirrhosis, alpha fetoprotein (AFP), aspartate aminotransferase (AST), and alanine aminotransferase (ALT) levels; the AST/ALT ratio was verified using the chart review.

### Selection of FGFR4 Polymorphisms

A total of four SNPs in FGFR4 were selected from the International HapMap Project data for this study. We included the non-synonymous SNPs rs351855 (Gly388Arg) and rs1966265 (Ile10Val) in the coding sequences of the gene. Furthermore, the SNP rs2011077 (in intron 11) and rs7708357 were selected in this study because the gene polymorphism of the SNP has been found to associate with prostate cancer [19, 20].

### Blood specimens

Whole blood specimens collected from the controls and patients with HCC were placed in tubes containing ethylenediaminetetraacetic acid (EDTA), centrifuged instantly, and stored at -80°C. The Institutional Review Board of Chung Shan Medical University Hospital approved this study, and informed written consent was obtained from each participant.

### DNA extraction

We collected whole blood samples from healthy controls and patients with HCC in tubes containing EDTA, which were centrifuged and subsequently stored at -80°C. The venous blood from each participant was drawn into Vacutainer tubes containing EDTA and stored at 4°C. Genomic DNA was extracted using QIAamp DNA blood mini kits (Qiagen, Valencia, USA) according to the manufacturer’s instructions, and the DNA was dissolved in TE buffer [10 mM Tris (pH 7.8) and 1 mM EDTA] and then quantitated using absorbance [optical density (OD) at 260 nm; OD260]. The final DNA prepared was stored at -20°C and used as templates for the following experiments.

### Quantitative real-time PCR

Allelic discrimination of the FGFR4 rs1966265, rs351855, rs2011077, and rs7708357 gene polymorphisms was assessed using an ABI StepOne real-time PCR system (Applied Biosystems), SDS V3.0 software (Applied Biosystems), and the TaqMan assay. The final volume for each reaction mixture was 5 mL, containing 2.5 mL TaqMan genotyping master mix, 0.125 mL TaqMan probe mix, and 10 ng genomic DNA. The reaction conditions included an initial denaturation step at 95 uC for 10 min followed by 40 cycles at 95°C for 15 s and 60°C for 1 min. For each assay, appropriate controls (nontemplate and known genotypes) were included in each typing run to monitor reagent contamination and as quality control. To validate results from the real-time PCR, approximately 5% of assays were repeated, and several cases of each genotype were confirmed using the DNA sequence analysis.

### Statistical analysis

The Hardy–Weinberg equilibrium was assessed using a chi-square goodness-of-fit test for biallelic markers. The distributions of demographic characteristics and genotype frequencies for different genotypes between the study participants and controls were analyzed using the chi-square test, and Fisher’s exact test was used for a small sample size for certain categories of variables. Student’s *t*-test was used to evaluate the differences in the laboratory findings between the 2 groups. The odds ratios (ORs) and their 95% confidence intervals (CIs) of the association between the genotype frequencies and HCC were estimated using multiple logistic regression models by controlling for covariates. A p value of less than 0.05 was considered statistically significant. The data were analyzed using SPSS 12.0 statistical software.

## Results

We found that 67.3% of healthy controls (595 in 884 participants) and 32.7% of patients with HCC (289 in 884 participants) consumed alcohol, and 37.1% of the healthy controls (221 in 595 participants) and 40.1% of patients with HCC (116 in 289 participants) smoked tobacco. No significant differences were observed in the distribution of alcohol consumption (p = 0.738) and tobacco use (p = 0.506) between healthy controls and patients with HCC, whereas age (controls: 51.00 ± 14.95; HCC: 63.02 ± 11.89; p< 0.001) and sex (p < 0.001) distributions between these 2 groups were significantly different (Table 1). To reduce the possible interference of confounding variables, the adjusted odds ratios (AORs) with 95% CIs were estimated using multiple logistic regression models after controlling for age and gender in each comparison.

**Table 1:** The distributions of the demographic characteristics of 595 controls and 289 patients with HCC.

Variable	Controls (N = 595)	Patients (N = 289)	p value
**Age (yrs)**	Mean ± S.D.	Mean ± S.D.	
	51.00 ± 14.95	63.02 ± 11.89	p<0.001*
**Gender**	n (%)	n (%)	
Male	487 (81.8%)	204 (70.6%)	
Female	108 (18.2%)	85 (29.4%)	p <0.001*
**Alcohol consumption**			
No	374 (62.9%)	185 (64.0%)	
Yes	221 (37.1%)	104 (36.0%)	p = 0.738
**Tobacco consumption**			
No	370 (62.2%)	173 (59.9%)	
Yes	225 (37.8%)	116 (40.1%)	p = 0.506
**Stage**			
I+II		191 (66.1%)	
III+IV		98 (33.9%)	
**Tumor T status**			
≤T2		194 (67.1%)	
>T2		95 (32.9%)	
**Lymph node status**			
N0		290 (96.9%)	
N1+N2		9 (3.1%)	
**Metastasis**			
M0		275 (95.2%)	
M1		14 (4.8%)	
**Child-Pugh grade**			
A		222 (76.8%)	
B or C		67 (23.2%)	
**Liver cirrhosis**			
Negative		63 (21.8%)	
Positive		226 (78.2%)	

Mann-Whitney U test or Fisher’s exact test was used between healthy controls and patients with HCC.

*, p value of < 0.05 was considered statistically significant.

Table 2 shows the genotype distributions and the association between HCC and FGFR4 polymorphisms. In controls, the frequencies of FGFR4 rs1966265 (χ^2^ value: 0.065), rs351855 (χ^2^ value: 2.997), rs2011077 (χ^2^ value: 0.002), and rs7708357 (χ^2^ value: 0.139) were in the Hardy–Weinberg equilibrium. The alleles with the highest distribution frequency at FGFR4 rs1966265, rs351855, rs2011077, and rs7708357 in patients with HCC and controls were heterozygous G/A, heterozygous C/T, heterozygous G/A, and homozygous G/G, respectively. AORs with their 95% CIs were estimated using multiple logistic regression models after controlling for age and sex with statistically nonsignificant differences between the patients with HCC and controls. Moreover, compared with the wild-type individuals, individuals with polymorphisms at rs1966265, rs351855, rs2011077, and rs7708357 showed no reduction in the risk of HCC in this study.

**Table 2:** The distribution frequency of FGFR4 genotypes in 595 healthy controls and 289 patients with HCC.

Variable	Controls (N = 595) n (%)	Patients (N = 289) n (%)	OR (95% CI)	AOR (95% CI)
**rs1966265**				
GG	151 (25.4%)	65 (22.5%)	1.00	1.00
GA	300 (50.4%)	160 (55.4%)	1.239 (0.875–1.755)	1.167 (0.782–1.743)
AA	144 (24.2%)	64 (22.1%)	1.032 (0.683–1.562)	0.845 (0.521–1.371)
GA+AA	444 (74.6%)	224 (77.5%)	1.172 (0.841–1.634)	1.063 (0.724–1.560)
**rs351855**				
CC (Gly/Gly)	159 (26.7%)	82 (28.4%)	1.00	1.00
CT (Gly/Arg)	314 (52.8%)	150 (51.9%)	0.926 (0.666–1.288)	0.994 (0.679–1.455)
TT (Arg/Arg)	122 (20.5%)	57 (19.7%)	0.906 (0.600–1.368)	1.039 (0.642–1.683)
CT+TT	436 (73.3%)	207 (71.6%)	0.921 (0.673–1.260)	1.005 (0.699–1.447)
**rs2011077**				
GG	147 (24.7%)	66 (22.8%)	1.00	1.00
GA	297 (49.9%)	159 (55.0%)	1.192 (0.842–1.689)	1.079 (0.720–1.616)
AA	151 (25.4%)	64 (22.1%)	0.944 (0.625–1.425)	0.773 (0.478–1.252)
GA+AA	448 (75.3%)	223 (77.2%)	1.109 (0.796–1.545)	0.977 (0.664–1.438)
**rs7708357**				
GG	577 (97.0%)	283 (97.9%)	1.00	1.00
GA	18 (3.0%)	5 (1.8%)	0.566 (0.208–1.541)	0.570 (0.190–1.707)
AA	0 (0%)	1 (0.3%)	——-	——
GA+AA	18 (3.0%)	6 (3.1%)	0.680 (0.267–1.731)	0.644 (0.225–1.840)

The odds ratios (ORs) with their 95% confidence intervals (CIs) were estimated using logistic regression models. The adjusted odds ratios (AORs) with their 95% Cis were estimated using multiple logistic regression models after controlling for age and sex. * p < 0.05 was considered statistically significant.

The distribution of the pathological status and FGFR4 genotypes in patients with HCC were estimated to clarify the role of FGFR4 polymorphisms in the pathological TNM stage of patients with HCC. Pathological status assessments included TNM staging, primary tumor size, lymph node involvement, and distant metastasis. No significant differences were observed between other FGFR4 genotypic frequencies and any pathological condition for the TNM stage variable (Table 3). Furthermore, the clinical status and FGFR4 genotypes in patients with HCC were evaluated to clarify the clinical role of FGFR4 polymorphisms in HCC, including the Child-Pugh grade, presence of an HBV or HCV infection, and liver cirrhosis. For FGFR4 rs351855, patients with the heterozygous G/A genotype had a high risk of HCC with cirrhosis (OR: 2.113, 95% CI: 1.188–3.831) (p < 0.01) compared with those with the homozygous G/G genotype, as shown in Table 4. No significant differences were observed between other FGFR genotypic frequencies and any clinical variable, except liver cirrhosis.

**Table 3:** Clinical TNM stage status and FGFR4 genotypic frequencies in 289 patients with HCC.

Variable	*FGFR4* rs1966265			*FGFR4* rs351855			*FGFR4* rs2011077		
	GG (N = 65)	GA+AA (N = 224)	p value	Gly/Gly (N = 82)	Gly/Arg + Arg/Arg (N = 207)	p value	GG (N = 66)	GA+AA (N = 223)	p value
**Clinical Stage**									
Stage I/II	40 (61.5%)	151 (67.4%)	0.379	55 (67.1%)	136 (65.7%)	0.824	41 (62.1%)	150 (67.3%)	0.438
Stage III/IV	25 (38.5%)	73 (32.6%)		27 (32.9%)	71 (34.3%)		25 (37.9%)	73 (32.7%)	
**Tumor size**									
≦ T2	41 (63.1%)	153 (68.3%)	0.430	55 (67.1%)	139 (67.1%)	0.990	42 (63.6%)	152 (68.2%)	0.492
> T2	24 (36.9%)	71 (31.7%)		27 (32.9%)	68 (32.9%)		24 (36.4%)	82 (31.8%)	
**Lymph node metastasis**									
No	61 (98.5%)	216 (96.4%)	0.406	78 (95.1%)	202 (97.6%)	0.277	65 (98.5%)	215 (96.4%)	0.395
Yes	1 (1.5%)	8 (3.6%)		4 (4.9%)	5 (2.4%)		1 (1.5%)	8 (3.6%)	
**Distant metastasis**									
No	60 (92.3%)	215 (96.0%)	0.224	80 (97.6%)	195 (94.2%)	0.231	61 (92.4%)	214 (96.0%)	0.239
**Yes**	5 (7.7%)	9 (4.0%)		2 (2.4%)	12 (5.8%)		5 (7.6%)	9 (4.0%)	

> T2: multiple tumors larger than 5 cm or tumor involving a major branch of the portal or hepatic vein(s).

**Table 4:** Liver cirrhosis status, virus status, and FGFR4 genotypic frequencies in 289 patients with HCC.

Variable	*FGFR4* rs1966265			*FGFR4* rs351855			*FGFR4* rs2011077		
	GG (N = 65)	GA+AA (N = 224)	p value	Gly/Gly (N = 82)	Gly/Arg + Arg/Arg (N = 207)	p value	GG (N = 66)	GA+AA (N = 223)	p value
**Child-Pugh grade**									
A	50 (76.9%)	172 (76.8%)	0.982	59 (72.0%)	163 (78.7%)	0.217	51 (77.3%)	171 (76.7%)	0.920
B or C	15 (23.1%)	52 (23.2%)		23 (28.0%)	44 (21.3%)		15 (22.7%)	52 (23.3%)	
**HBsAg**									
Negative	32 (49.2%)	139 (62.1%)	0.064	51 (62.2%)	120 (58.0%)	0.510	33 (50.0%)	138 (61.9%)	0.084
Positive	33 (50.8%)	85 (37.9%)		31 (37.8%)	87 (42.0%)		33 (50.0%)	85 (38.1%)	
**Anti-HCV**									
Negative	32 (49.2%)	115 (51.3%)	0.765	46 (56.1%)	101 (48.8%)	0.263	33 (50.0%)	114 (51.1%)	0.873
Positive	33 (50.8%)	109 (48.7%)		36 (43.9%)	106 (51.2%)		33 (50.0%)	109 (48.9%)	
**Liver cirrhosis**									
Negative	16 (24.6%)	47 (21.0%)	0.532	26 (31.7%)	37 (17.9%)	0.010*	16 (24.2%)	47 (21.1%)	0.584
Positive	49 (75.4%)	177 (79.0%)		56 (68.3%)	170 (82.1%)		50 (75.8%)	176 (78.9%)	

**Fig 1:**
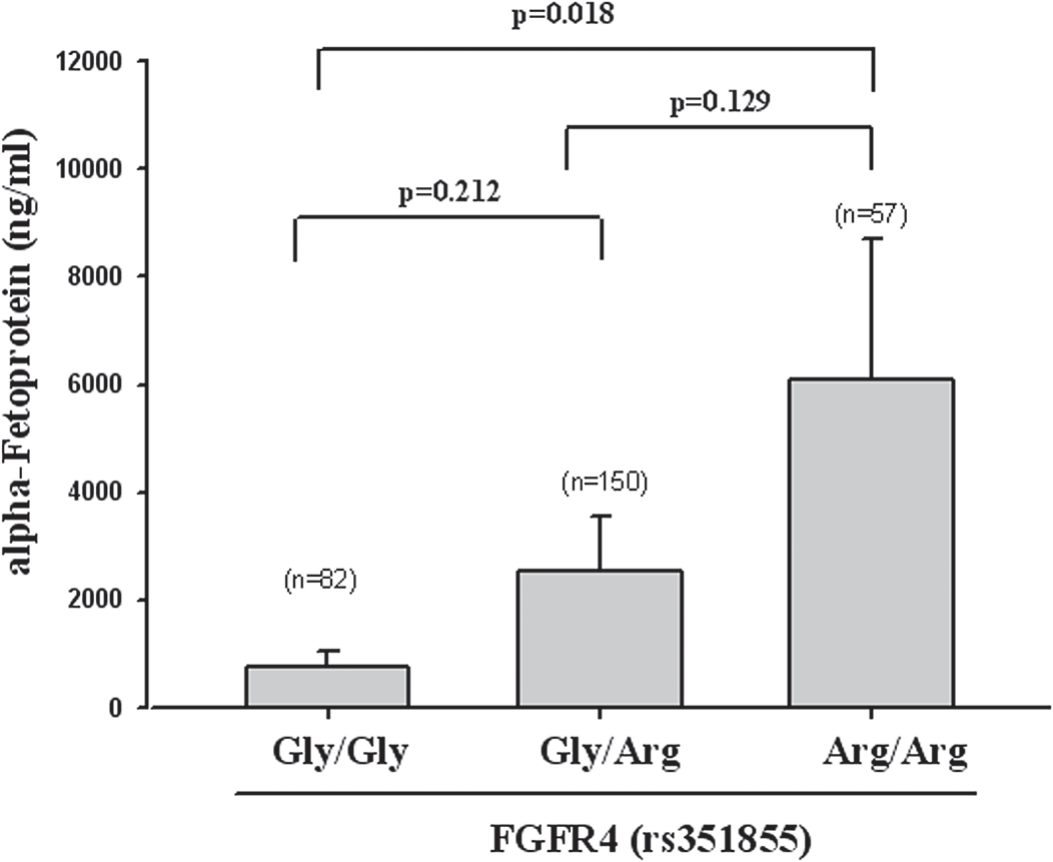
The AFP level of the homozygous Arg/Arg genotype of FGFR4 rs351855 compared with that of the Gly/Gly genotype (p = 0.018).

Table 5 illustrates the association of FGFR4 genotypic rs351855 frequencies with the HCC laboratory findings of AST, ALT, and the AST/ALT ratio. Overall, the AST/ALT ratio had a statistically significant association between the variant genotypes CC and CT + TT in patients with HCC (p < 0.001). Moreover, the homozygous Arg/Arg genotype of FGFR4 rs351855 was associated with a high AFP level when compared with the Gly/Gly genotype (p = 0.018), as illustrated in Fig 1.

**Table 5:** Association of FGFR4 genotypic frequencies with the HCC laboratory findings.

Characteristic	AST ^a^ (IU/L)	ALT ^a^ (IU/L)	AST/ALT ratio ^a^
**rs1966265**			
GG	168.98 ± 48.71	168.51 ± 41.17	1.18 ± 0.06
GA+AA	145.96 ± 19.10	114.49 ± 14.20	1.61 ± 0.12
p value	0.602	0.118	0.053
**rs351855**			
CC	175.57 ± 38.88	100.84 ± 13.39	1.98 ± 0.29
CT+TT	141.46 ± 20.56	136.86 ± 19.37	1.33 ± 0.05
p value	0.404	0.260	0.001*
**rs2011077**			
GG	167.39 ± 47.99	166.70 ± 40.58	1.19 ± 0.06
GA+AA	146.33 ± 19.19	114.78 ± 14.26	1.61 ± 0.12
p value	0.631	0.131	0.052
**rs7708357**			
GG	153.52 ± 18.75	128.66 ± 14.69	1.51 ± 0.09
GA+AA	39.00 ± 8.39	31.50 ± 10.53	1.47 ± 0.28
p value	0.375	0.337	0.942

## Discussion

HCC is one of the most common and lethal malignancies in the world; preventing its occurrence and reducing HCC-related mortality should be the primary aim in public healthcare. HBV or HCV infections, habitual alcohol consumption, history of liver cirrhosis, and a family history of HCC are the common major etiologies for HCC in Taiwan [22–24]. The data in Table 1 indicate that the ratios of tobacco consumer/nonconsumer in control participants (37.8:62.2) and in patients with HCC (40.1:59.9) were almost equal. Moreover, the percentage of alcohol consumer in patients with HCC (36.0%) was approximately equal to that in controls (37.1%). This result implies that the risk of HCC is not linked to smoking and alcohol consumption. Hepatitis virus infections are a highly endemic factor for HCC in Asian countries. The infection may increase oxidative stress in hepatic cells and result in DNA changes and instability, thus increasing the risk of cirrhosis and/or HCC. Many studies have reported that HBV and HCV infections are among the most frequent causes for HCC in Asia [25–28]. In the present study, HBV and HCV infections were present in the patients with HCC; however, the frequencies of HBV- and HCV-positive cases were irrelevant to the presence of FGFR4 genotypes (Table 4). This indicates that the sensitivity of HBV and HCV infections in patients with HCC with polymorphic rs1966265, rs351855, rs2011077, and rs7708357 genotypes is not different from that observed in patients with the ancestral genotype.

FGFRs consist of 4 closely related genes (FGFR1-4) that are associated with the development of several types of human cancers such as breast, pulmonary, gastric, esophageal, salivary gland, bladder, and ovarian cancer[29–32]. Moreover, studies on FGFR4, including its high expression in the liver and its role in regulating hepatobiliary functions and in developing HCC, are increasing. The study of Tsou et al. indicated that multiple kinases including PDGFR-b, FGFR-4, and Axl are activated in different tumors and confirmed the existence of molecular heterogeneity in the mechanisms sustaining autonomous cell growth in liver tumor formation [33]. Ansell et al demonstrated a substantial association between FGFR4 Gly388Arg polymorphism and the risk of breast and prostate cancer in Asians caused by changes in the components of the intracellular signal transduction pathways [34]. However, few studies have reported the expression of FGFR4 and its associated gene polymorphisms with HCC. In our study, we evaluated the FGFR4 protein expression as a risk indicator for HCC, which showed no association with rs1966265, rs351855, rs2011077, and rs7708357 (Table 2). In addition, Gly388 was not the associated allele in HCC; this is similar to the results of the study by Yang et al [35].

We observed that genetic polymorphism in FGFR4 rs351855 (Gly/Arg and Arg/Arg) was associated with high risk (OR: 2.113, 95% CI: 1.188–3.831) of liver cirrhosis and markedly increased the AFP level in patients with HCC. FGFR4 rs351855 polymorphisms may lead to alterations in the coding sequence of the encoded protein, thereby impacting the initiation and/or progression in HCC, particularly in patients with liver cirrhosis [33]. Moreover, Ho et al confirmed that FGFR4 contributes substantially to HCC progression by modulating AFP secretion, proliferation, and antiapoptosis. Frequent overexpression of FGFR4 in patients renders its inhibition as a novel and much needed pharmacological approach against HCC [15]. In our study, we observed that FGFR4 SNPs appeared to modulate tumor progression without the presence of any significant risk factors in HCC. Further research on FGFR4 gene polymorphisms is necessary to evaluate the mechanisms and pathways.

FGFR4 contributes to the limitation of the resultant liver fibrosis [36]. The AST/ALT ratio has been used to assess the severity of disease in patients with chronic liver disease or to predict HBV-related HCC risk [37, 38]. Our results indicate that the AST/ALT ratio in the FGFR4 genotype at rs351855 is significantly different (p < 0.01) in CC composition with CT + TT. This finding suggests that the increase in the AST/ALT ratio in genetic polymorphism of FGFR4 at rs351855 is associated with its early detection in HCC. Some researchers have reported that FGFR4 contributes substantially to HCC progression by modulating AFP secretion [15]. Our results showed that FGFR4 rs351855 was associated with a high level of AFP secretion. Therefore, the increase in serum AFP level is significantly different in Arg/Arg (6076.12 ± 2629.01) when compared with Gly/Gly (778.55 ± 25.84) (p = 0.018), and this could be another marker for early diagnosis of HCC on the basis of genetic polymorphism of FGFR4 at rs351855.

Moreover, we found that FGFR4 had no significant correlation with other prognostic factors such as HBV and HCV infections, Child-Pugh grade, tumor size, lymph node metastasis, and distant metastasis (p > 0.05); these results were identical to those of the study by Chen et al [39]. In addition, hepatocyte FGFR4 has been reported to potentially play a crucial role in the orderly restoration of liver mass and morphology, independent of hepatocyte proliferation, following acute and chronic toxic insults to the liver [36]. In this study, we found that FGFR4 polymorphisms had no correlation with clinical stage parameters of TNM. We reported that FGFR4 SNPs appeared to modulate tumor progression without major cancer risk factors in HCC.

In conclusion, the study findings show a significant association between FGFR4 polymorphisms and liver cirrhosis in HCC. A variant FGFR4 allele may be an indicator for HCC in liver cirrhosis, the AST/ALT ratio, and the AFP level. We concluded that FGFR4 SNPs at rs351855 can be a useful marker for predicting the liver cirrhosis status in HCC. Moreover, this is the first study that correlated liver cirrhosis, the AST/ALT ratio, and the AFP level caused by FGFR4 polymorphic variants and their association with HCC.
